# The Influence of Different Garlic Genotypes on Yogurt Production

**DOI:** 10.1002/fsn3.4606

**Published:** 2025-03-04

**Authors:** H. Ceren Akal, Gökçe Eminoğlu, Selen Akan

**Affiliations:** ^1^ Department of Dairy Technology Ankara University Faculty of Agriculture Ankara Turkey; ^2^ Department of Horticulture Ankara University Faculty of Agriculture Ankara Turkey

**Keywords:** functional, garlic, volatile, yogurt

## Abstract

Genotypic differences influence many characteristics of garlic. These differences are particularly prominent in antioxidant, phenolic, and volatile compounds. Therefore, garlics obtained from four different regions of Turkey (Ankara, Mersin, Maraş, Taşköprü) were used in yogurt production. Gross composition, acidity, antioxidant capacity, phenolic compounds, water‐holding capacity, volatile compound profile, microbiological, textural, and sensory properties of the samples were determined during 30‐day storage period. The addition of garlic did not significantly change the composition, acidity, water‐holding capacity, and textural properties of the samples. However, the count of lactic acid bacteria was lower in the samples with garlic compared to the control sample. In addition, depending on the garlic genotype, the addition of garlic increased the antioxidant and phenolic contents of yogurts at varying levels. Yogurt with Taşköprü garlic (sample coded D) showed the highest phenolic compound and antioxidant capacity. Similarly, sulfur compounds were detected in garlic‐added yogurts at varying levels depending on the genotype. Among these compounds, diallyl disulfide was found to be at the highest level. All sulfur compounds were found at the highest levels in yogurt with Ankara and Taşköprü garlics. These yogurt samples (coded samples A and D), which were identified with high amounts of diallyl sulfide, diallyl disulfide, and diallyl trisulfide, received significantly lower taste scores from the panelists compared to the control sample due to their strong and pungent taste. This study revealed that garlic obtained from different genotypes had different effects on yogurt properties.

## Graphical abstract

Genotypic differences influence many characteristics of garlic. These differences are particularly prominent in antioxidant, phenolic, and volatile compounds. Therefore, garlic obtained from four different regions of Turkey (Ankara, Mersin, Maraş, Taşköprü) was used in yogurt production.

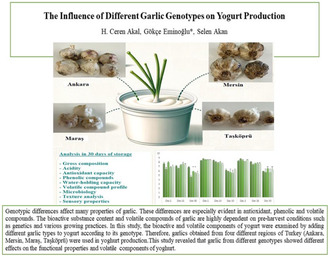

## 1 Introduction

In recent years, we have seen an increasing trend of consumers seeking healthier and more beneficial foods. This trend has become even more pronounced in light of the COVID‐19 outbreak, with a notable shift in eating habits towards functional foods. These are foods that not only provide essential nutrients but also offer additional health benefits. As such, strategies like diversification of use for functional foods or introducing functionality to foods that are already widely consumed can be an effective approach.

Garlic is one such functional food, known for its numerous beneficial properties. Different studies have highlighted the health effects of garlic. These include antiviral (Rouf et al. 2020), antifungal (Khounganian et al. 2023), antibacterial (Bhatwalkar et al. 2021), neuroprotective (Farooqui and Farooqui 2018), wound healing assistance (Shokouhi Sabet Jalali et al. 2009), anti‐thrombotic (Eric Block et al. 2017), anti‐diabetic (Saikat et al. 2022), and anti‐atherosclerotic benefits (Sobenin et al. 2019). Furthermore, garlic possesses pharmaceutical potential due to its therapeutic value in treating various disorders (Singh et al. 2020). Garlic's health benefits are linked to its various sulfur compounds, including allicin, ajoene, vinyl‐dithiin, and other volatile organosulfur compounds, all of which are metabolized from alliin (Verma, Aggarwal et al. 2023). However, its sharp taste and strong odor can limit its consumption if used alone. To overcome this, consumers have started to incorporate garlic into various foods, not only to mask its intense flavor but also to add a new dimension to the taste and aroma of their meals. This approach offers a sensory innovation while also introducing the functional benefits of garlic to a wider range of foods. Garlic has been tried in various foods, from shrimp head (Rujirapong et al. 2022), pork (Leong et al. 2010), olive oil (Kishimoto and Kashiwagi 2020), bread (Kairam et al. 2021), to soy products (Woo et al. 2016). In addition to these studies (Verma, Dey 2023). A study was conducted on the production of snacks containing garlic paste and it was reported that samples containing 10% garlic paste were the most appreciated samples in terms of sensory evaluation.

Yogurt, a dairy product widely consumed and revered for its health benefits, has also seen the introduction of garlic. The effect of both imported and local garlic on the quality of yogurt was researched (Arslaner 2020). Another study explored the *Staphylococcus aureus* inhibitory properties of yogurt produced with the addition of garlic juice (S. G. Lee et al. 2009). The sensory properties of yogurt with the addition of black garlic were also a subject of research (Priadi et al. 2023). Despite these, a gap in research was found regarding the effect of garlic from different geographical origins on yogurt production.

Numerous researches have indicated that different genotypes of garlic can affect many properties of the plant, particularly the antioxidant phenolic contents (Akan, Yarali Karakan, and Horzum 2022; Avgeri et al. 2020; Hornícková et al. 2010; Petropoulos et al. 2018). Turkey, with its diverse climates and agricultural areas due to its unique geographical location, is home to a rich variety of garlic genotypes. This vast array of genotypes was the focus of studies conducted by Akan (2022) and Akan et al. (2022), which investigated their genotypic characteristics. In these studies, garlic was harvested from different geographical regions of Turkey, its morphological properties were measured, and volatile compounds, antioxidant, and phenolic compounds were determined.

Building on this, the current study explored the potential use of garlic from four different Turkish regions (Ankara, Mersin, Maraş, Taşköprü), all with distinct genotypic characteristics. The aim is not only to understand the potential impact of these different garlic genotypes on yogurt production but also to determine whether these genotypic differences translate into tangible differences in the properties of the end product. In doing so, it hopes to contribute to the growing body of knowledge on the functional benefits of garlic and its potential applications in the food industry.

## 2 Materials and Methods

### 2.1 Materials

The bovine milk used for yogurt production was sourced from Ankara University. The starter culture, including a mixture of *Str. thermophilus* and *Lb*. *delbrueckii* subsp. *bulgaricus*, was provided by Chr. Hansen. This study also utilized four types of garlic from different regions in Turkey: Ankara, Mersin, Maraş, and Taşköprü. The genotypic features of these garlic varieties were previously identified in a study by S. Akan (2022).

### 2.2 Yogurt Production

The dry matter content of the raw milk was standardized to 16% by adding skimmed milk powder. The standardized milk was then homogenized at 70°C by using ultra‐turrax. After homogenization, the milk was heated to 90°C for 5 min and then quickly cooled to 45°C. At this point, 2% (w/v) of the yogurt starter culture (CH‐1, Chr Hasen, Denmark) was added. The milk was incubated at 43°C until the pH decreased to 4.0, a process that took about 4 h. Fermentation was stopped by cooling the mixture to 4°C, producing 5 kg of yogurt in each batch.

**FIGURE 1:**
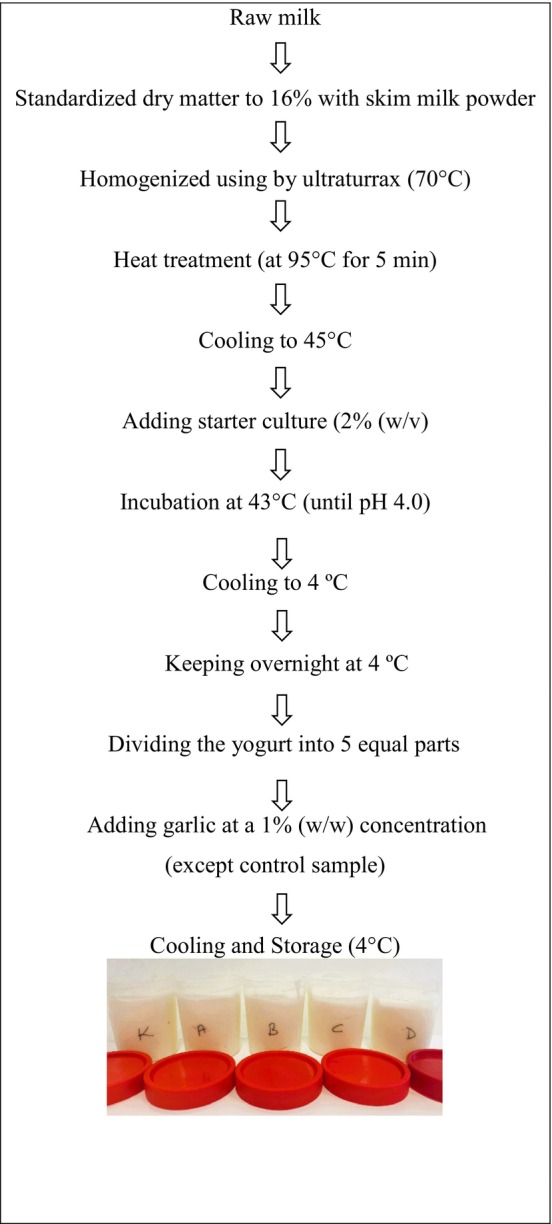
Flow diagram of yogurt production.

Next, the yogurt clot was mixed and divided into five equal parts. Garlic, prepared by crushing, was added at a 1% (w/w) concentration to four of these portions. The fifth portion was prepared without garlic as a control sample (Figure 1). This process was replicated twice. Each yogurt sample was stored at 4°C, and analyses were conducted on days 1, 15, and 30.

### 2.3 Gross Composition

The total dry matter and ash in the samples were determined using the gravimetric method, as described by the IDF (2005). Fat content was measured as a percentage using the Gerber method, which uses a milk butyrometer. The total protein content was quantified using the Kjeldahl method, as detailed in the IDF (1993). A digital pH meter (Mettler‐Toledo) with a combined electrode was used for this analysis.

### 2.4 Microbiological Analysis

To count *Str. thermophilus* and *Lb. delbrueckii* subsp. *bulgaricus*, we first diluted the samples with Ringer's solution. We inoculated the diluted samples on M‐17 Agar (Merck) for *Str. thermophilus*, and on MRS (de Man Rogosa Sharpe) Agar (Merck) for *Lb. delbrueckii* subsp. *bulgaricus*. After inoculation, we incubated the petri dishes under specific conditions: for *Str. thermophilus*, at 37°C for 48 h in an aerobic environment, and for *Lb. delbrueckii* subsp. *bulgaricus*, in an anaerobic environment. After the incubation period, we counted the colonies that had formed.

### 2.5 Total Phenolic and Antioxidant Capacity Analysis

For both analyses, 10 g sample was prepared by centrifuging at 12000 rpm for 10 min. To determine phenolic matter, 1 mL of the supernatant was mixed with 95% ethanol, 5 mL distilled water, and 0.55 mL of 50% Folin–Ciocalteu reagent. After standing for 5 min, 5% Na_2_CO_3_ was added, and the solution was left in the dark for 1 h. The absorbance was then measured at 725 nm using a spectrophotometer (Lambda 25 UV/Vis, PerkinElmer, Singapore) The samples' phenolic content was calculated as gallic acid equivalence, following the method described by Apostolidis, Kwon, and Shetty (2006).

For total antioxidant capacity, 250 μL of the supernatant (filtered through Whatman 40 filter paper) was used. This was mixed with 1500 μL of 0.1 mM DPPH solution and left in the dark for 30 min. The absorbance was then measured at 517 nm using a spectrophotometer (Lambda 25 UV/Vis, PerkinElmer, Singapore) The percentage inhibition value was calculated using the given equation.

$$\%\text{Inhibition}=\frac{\;A_{b}-A_{s}}{A_{b.}}$$

where*Ab*: Blank absorbance value*As*: Absorbance value of the sample.

### 2.6 Water‐Holding Capacity

To determine the water‐holding capacity (WHC) of garlic yogurt samples, 25 g samples were centrifuged (Sigma 3‐18 K, Sartorius AG, Gottingen, Germany) at 10000 rpm for 15 min. The WHC value was calculated using the equation given below (Isanga and Zhang 2009).

$$\mathrm{WHC}\%=1-\frac{w_{c}}{w_{s}}\;x\;100$$

where*W*_*c*_: Weight after centrifugation,*W*_*S*_: The sample weight.

### 2.7 Determination of Textural Properties

The texture of the yogurt samples was analyzed using the back extrusion method, with a TA‐XT Plus Texture Analyzer (Stable Micro Systems, UK). A 5 kg load cell and a 35 mm probe were used for this process. The firmness, consistency, cohesiveness, and viscosity index were determined at a consistent speed of 1.0 mm/s, aiming for a distance of 10 mm. This method enabled precise quantification of the garlic yogurt samples' essential textural features.

### 2.8 Determination of Volatile Profile

The volatile profile of the yogurt samples was determined using the Solid Phase Micro Extraction (SPME) method. A 5 g sample was placed into 20 mL vials, and 10 μL of an internal standard was added. This standard was composed of 8.1 ppm of 2‐methyl‐3‐heptanone and 2‐methyl pentanoic acid in methanol. The mixture was then agitated at 50°C for 30 min. After this, the fiber was attached and kept at 50°C for an extra 30 min, enabling the volatile aroma components to stick to the fiber.

The aroma components were analyzed using a gas chromatography–mass spectrometry (GC–MS) system, specifically the Agilent 7890A GC‐5975 MSD. Helium gas was used as the carrier at a flow rate of 1.0 mL/min. The column used was a DB‐Wax (30 m, 0.25 mm, 0.25 μm) column (J. H. Lee et al. 2003). The specific oven temperature program used for the aroma profile analysis was tailored to optimize the separation and identification of the volatile compounds in the samples.

**Table:** 

Increase	Temperature	Waiting
—	40°C	10 min
5°C/min	110°C	—
10°C/min	250°C	10 min

Flavor components in the yogurt samples were quantified by analyzing peak areas from GC–MS. Component identification was aided by the Wiley, NIST, and Flavor libraries in the GC–MS system. The volatile component amounts were calculated in relation to the standard substances used in the analysis.

### 2.9 Determination of Sensorial Properties

For the sensory evaluation, a panel of seven individuals (4 women‐3 men, aged 37–50 years) was assembled. They were given the yogurt samples at the storage temperature of 4°C. A scoring test with a nine‐point scale (9; like very much, 1; dislike very much) was used to evaluate the samples (Clark and Costello 2016). This allowed the panelists to assess various sensory aspects of the yogurt, including taste, aroma, texture, and overall acceptability, creating a comprehensive sensory profile of the product.

### 2.10 Statistical Analysis

Statistical analyses of the data were performed using Minitab‐19 package program (Minitab Inc. State College, PA). Analysis of variance (ANOVA) was carried out to determine statistical differences among yogurt samples and storage times. Tukey's multiple range test was applied for the identification of statistically significant differences; *p* < 0.05 was accepted as the significance level.

## 3 Results and Discussion

### 3.1 Gross Composition

The composition of yogurt samples is presented in Table 1. The fat, protein, dry matter, and ash values of yogurt samples ranged between 3.50%–3.62%, 4.82%–4.94%, 16.92%–17.26%, and 1.08%–1.16%, respectively. It was determined that the addition of garlic from different genotypes did not affect the gross composition of the yogurts. Since the amount of garlic added was low (1%), it did not cause a significant change in the dry matter and ash values (*p* > 0.05). Similarly, Gündoğdu, Cakmakci, and Dagdemir (2009) showed that the addition of garlic to yogurt did not affect the composition of yogurt.

**TABLE 1:** Gross composition of yogurt samples on day 1(mean ± SE).

	Fat	Protein	Dry matter	Ash
K	3.50 ± 0.00	4.82 ± 0.04	16.92 ± 0.05	1.16 ± 0.01
A	3.62 ± 0.02	4.86 ± 0.04	16.97 ± 0.21	1.17 ± 0.02
B	3.55 ± 0.05	4.94 ± 0.01	17.07 ± 0.06	1.09 ± 0.02
C	3.60 ± 0.00	4.89 ± 0.00	17.26 ± 0.56	1.08 ± 0.00
D	3.60 ± 0.00	4.94 ± 0.01	16.96 ± 0.23	1.08 ± 0.00

*Note:* K: Control sample (not containing garlic), A: Yogurt containing Ankara garlic, B: Yogurt containing Mersin garlic C: Yogurt containing Maraş garlic, D: Yogurt containing Taşköprü garlic Non‐lettering columns indicates the differences between scores are not found significant (*p* > 0.05).

Abbreviation: SE, standard error.

### 3.2 Titratable Acidity and pH Values

**FIGURE 2:**
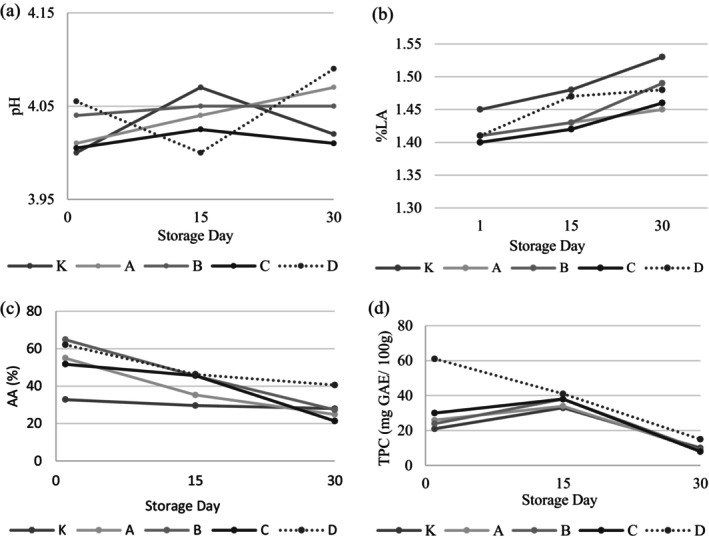
(a) pH, (b) Lactic acid, (c) Antioxidant activity (AA), and (d) Total phenolics content (TPC) of yogurt samples. K: Control sample (not containing garlic), A: Yogurt containing Ankara garlic, B: Yogurt containing Mersin garlic C: Yogurt containing Maraş garlic, D: Yogurt containing Taşköprü garlic.

The acidity values of yogurt samples are given in Figure 2. The pH values of yogurt samples ranged from 4.00 to 4.09, while the titratable acidity values varied between 1.40% and 1.53% lactic acid. There was no significant difference in acidity values among the samples. The acidity values of the samples slightly increased during the storage period. However, a significant relationship between storage time and acidity values could not be found (*p* > 0.05). Similarly, Shori and Baba (2014) added garlic extract to the yogurts produced from cow milk and reported that the addition of garlic did not cause a significant change in the pH value of the yogurt.

### 3.3 Antioxidant Capacity and Phenolic Compounds

The health benefits of garlic are attributed to its bioactive compounds, especially organosulfur compounds responsible for its sharp taste. Additionally, it is also known that garlic contains high amounts of phenolic compounds (Shang et al. 2019). Garlic is one of the products with the highest total phenolic content among commonly consumed plant products (Vinson et al. 1998). The antioxidant capacity (AC) and total phenolic component (TPC) values of yogurt samples are shown in Figure 2 and Table S1. The AC of yogurt samples with garlic of different genotypes were found to be higher than those of the control sample (*p* < 0.05). During the storage period, the AC of the garlic‐added yogurt samples ranged from 21.36% to 62.07% while the AC content of control sample ranged from 27.98% to 32.82%. Similarly, in a study conducted by Shori and Baba (2014) yogurt samples with garlic powder showed a significant increase in antioxidant capacity and phenolic compound. It is known that garlic contains high levels of antioxidative and phenolic components (Toledano‐Medina et al. 2016; Upadhyay 2017). The sample with the highest phenolic component and antioxidant capacity among the samples was Taşköprü garlic‐added yogurt (D‐coded sample). Akan et al. (2022) determined in their study with different genotypes of garlic that Taşköprü garlic had high phenolic content and antioxidant activity. In the same study, it was determined that the TPC and AC of garlic grown in Turkey, which were also used in our study, were 34.88–24.67 mg GAE/100 g and 75.33%–48.33%, respectively.

The AC values of yogurt samples decreased during storage. This can be attributed to the decrease in antioxidant components in garlic during storage (Akan et al. 2022). However, degradation or oxidation of the antioxidant components in the yogurt composition may have caused a decrease in antioxidant capacity during storage (Starowicz and Zieliński 2019). Although the control sample does not contain garlic, the TPC content was found to be high (10–33 mg GAE/100 g). This may be due to the dairy animal being fed with high phenolic compound‐containing feed (Shori 2020) or as a result of the amino acid catabolism of lactic acid bacteria (O'Connell and Fox 2001). The analysis results indicated that storage time had a significant effect on the levels of AOC and TPC (*p* < 0.05). The TPC values of the samples significantly decreased at the end of storage (*p* < 0.05).

Additionally, in terms of the effect of garlic addition on the composition, TPC and AC of yogurt, yogurt samples with garlic addition had higher fat, protein, dry matter and higher AC and TPC contents compared to the control sample.

### 3.4 Microbiological Properties

**FIGURE 3:**
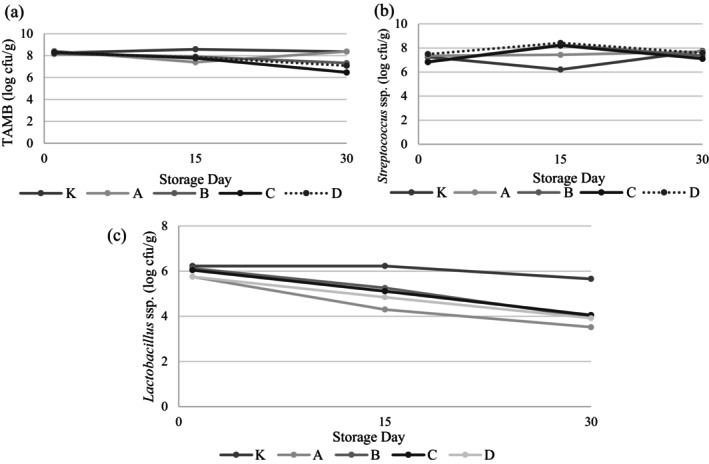
Microbiological properties on yogurt samples (log cfu/g). K: Control sample (not containing garlic), A: Yogurt containing Ankara garlic, B: Yogurt containing Mersin garlic C: Yogurt containing Maraş garlic, D: Yogurt containing Taşköprü garlic.

Microbiological properties of yogurt samples are present in Figure 3 and Table S2. Interactions between storage days and samples were detected in the total mesophilic aerobic bacteria (TMAB), *Streptococcus* ssp., and *Lactobacillus* ssp. contents of the samples (*p* < 0.05). Except for Sample C, no significant change was observed in the TMAB counts of yogurt samples during storage (*p* > 0.05). A significant decrease was observed in Sample C on the 30th day of storage. No difference was observed among the samples in terms of *Streptococcus* ssp. counts. Over the storage period, an increase in *Streptococcus* counts was observed in all samples on day 15, followed by a decrease on day 30. In terms of *Streptococcus* spp., samples coded B and C contain lower numbers of bacteria than the control sample on the storage day 1. A study conducted by Arslaner (2020) also reported that the addition of garlic reduced the number of yogurt bacteria compared to the control sample. The same effect is observed more significantly in the count of *Lactobacillus* ssp. It has been revealed that garlic‐added samples contained lower amounts of *Lactobacillus* ssp. than the control sample for all storage days. Especially as the storage progressed, this difference increased further, and on the 30th day of storage, the count of *Lactobacillus* ssp. in all garlic‐added samples was significantly lower than that in the control sample (*p* < 0.05). Again, in the study conducted by Arslaner (2020) it was reported that the addition of 1% garlic significantly reduced the number of bacteria. It is thought that the bacteriostatic effect of garlic on yogurt bacteria is due to the fact that the allicin contained in garlic prevents the growth of bacteria by changing the sulfhydryl groups necessary for cell proliferation (Block et al. 2010).

### 3.5 Textural Properties and WHC

**FIGURE 4:**
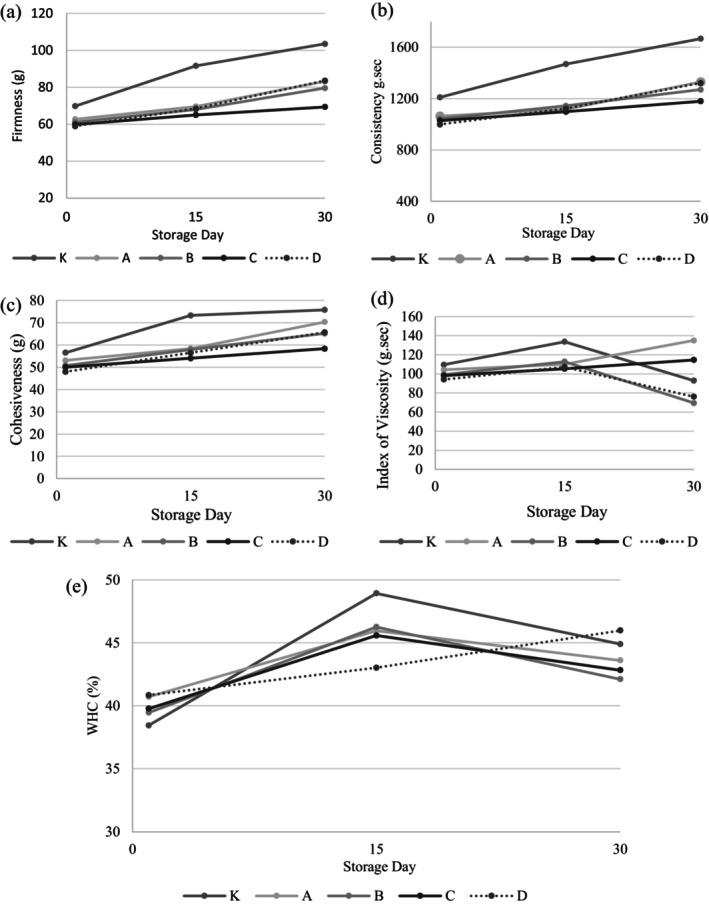
Textural properties and WHC values of yogurt samples. K: Control sample (not containing garlic), A: Yogurt containing Ankara garlic, B: Yogurt containing Mersin garlic C: Yogurt containing Maraş garlic, D: Yogurt containing Taşköprü garlic.

Textural properties and WHC results of yogurt samples are given in Figure 4 and Table S3. Tiwari et al. (2021) reported that when polysaccharide compounds are introduced into yogurt, there is a notable increase in both the WHC and hardness values. This is mainly due to the synergistic interaction between the polysaccharides and casein present in the yogurt. In contrast, during our research, we found that despite its high carbohydrate (Yusuf et al. 2018) the addition of garlic did not result in any significant alterations to the yogurt's WHC or hardness as compared to the control sample (*p* > 0.05). This unexpected outcome can be attributed to a couple of factors: firstly, both the samples used in the study were stirred types of yogurt. Secondly, the quantity of garlic added to the yogurt was minimal, which could explain the lack of a significant effect. When we examined the effects of both the samples and the storage days on the cohesiveness and index of viscosity values, we found no significant effect (*p* > 0.05). However, upon analyzing the storage days for concentration, WHC, and hardness, a significant difference (*p* < 0.05) was detected, with all these values demonstrating an increase as the storage period progressed.

As stated by Tamime and Robinson (2000), an increase in acidity promotes the WHC of milk proteins. This, in turn, influences the overall hardness of the yogurt. Interestingly, throughout the storage period of our samples, while the pH values remained relatively consistent, there was a significant increase in titration acidity values after 30 days (as observed in Figure 2). However, when comparing the acidity values, WHC, and hardness values of the yogurt samples on day 30, no significant difference was observed (*p* > 0.05). This suggests that the duration of storage and changes in acidity may not necessarily correlate to changes in these parameters.

Garlic, regardless of its genotypic characteristics, does not significantly affect the physical properties of yogurt. Only one textural property, the consistency value, showed a significant difference (*p* < 0.05) between samples with garlic and the control sample. This value, derived from the positive area of the graph in texture determination, reflects various rheological properties of the food, such as viscosity, cohesion, and surface tension (Wagner et al. 2017). The addition of garlic did not significantly affect the hardness, cohesiveness, or viscosity index of the yogurt. However, it did create a difference in the viscosity value compared to the control, with the consistency of yogurt containing garlic being significantly lower than that of the control sample.

### 3.6 Volatile Compounds

In present study, forty‐three compounds were identified in the volatile fractions of yogurt samples, including 8 acids, 4 alcohols, 12 aldehydes and ketones, 5 esters and 14 sulfur compounds and this compounds are listed by chemical groups in Table 2 and Table S4–S8.

**TABLE 2:** Volatile compounds in yogurt samples during 30 days of storage (μg/100 g) (mean ± SE).

Carboxylic acids^a^	Days	K	A	B	C	D
Octanoic acid	1	1.38 ± 0.04	57.94 ± 2.50	3.34 ± 0.50	93.12 ± 2.53	120.72 ± 5.65
	15	72.55 ± 1.22	53.89 ± 2.30	83.72 ± 0.56	87.59 ± 5.06	103.07 ± 8.84
	30	24.26 ± 3.23	52.04 ± 0.18	159.67 ± 2.32	58.67 ± 0.12	33.43 ± 0.84
Hexanoic acid	1	N.D.	141.44 ± 13.15	5.45 ± 0.53	124.42 ± 3.30	251.57 ± 7.70
	15	N.D.	165.16 ± 8.79	114.31 ± 2.50	137.32 ± 1.64	242.91 ± 3.58
	30	23.53 ± 2.49	N.D.	163.25 ± 2.08	35.84 ± 0.21	34.11 ± 2.88
Acetic acid	1	2.52 ± 0.32	81.90 ± 5.14	3.24 ± 0.71	87.58 ± 2.06	137.09 ± 0.86
	15	112.52 ± 4.85	114.94 ± 8.91	53.78 ± 1.26	55.11 ± 1.83	147.37 ± 10.35
	30	44.23 ± 0.99	38.58 ± 3.35	93.99 ± 1.98	58.20 ± 0.63	37.45 ± 0.64
Butyric acid	1	1.72 ± 0.06	73.72 ± 14.24	1.79 ± 0.25	35.83 ± 0.86	72.17 ± 4.37
	15	54.83 ± 4.29	58.24 ± 3.40	28.52 ± 1.32	27.57 ± 0.80	73.47 ± 8.26
	30	23.23 ± 2.28	23.25 ± 3.40	95.96 ± 1.91	32.28 ± 0.81	20.16 ± 1.74
Aldehydes						
Acetaldehyde	1	0.26 ± 0.03	1.75 ± 0.08	0.71 ± 0.04	1.55 ± 0.40	1.52 ± 0.38
	15	N.D.	N.D.	N.D.	N.D.	N.D.
	30	N.D.	N.D.	N.D.	N.D.	N.D.
Acetone (2‐Propanone)	1	0.48 ± 0.04	27.02 ± 2.12	1.43 ± 0.03	22.52 ± 1.67	78.97 ± 11.52
	15	2.24 ± 0.05	29.01 ± 3.04	8.69 ± 0.3	14.54 ± 1.44	35.45 ± 4.05
	30	5.99 ± 0.16	10.45 ± 0.61	9.00 ± 0.26	13.75 ± 2.16	16.86 ± 0.06
Ketones						
2‐Butanone	1	0.33 ± 0.22	N.D.	0.42 ± 0.10	N.D.	4.82 ± 0.50
	15	0.30 ± 0.03	6.47 ± 1.20	7.66 ± 0.51	6.95 ± 0.20	N.D.
	30	1.55 ± 0.53	4.45 ± 1.28	12.56 ± 0.36	N.D.	4.63 ± 0.55
2.3‐Butanedione (diacetyl)	1	0.58 ± 0.04	11.05 ± 1.99	0.50 ± 0.08	9.35 ± 0.36	16.40 ± 2.13
	15	8.72 ± 0.27	7.65 ± 1.85	5.06 ± 0.40	7.83 ± 0.15	10.82 ± 0.35
	30	N.D.	N.D.	N.D.	N.D.	N.D.
Esters						
Butyl butyrate	1	0.57 ± 0.15	3.47 ± 0.23	0.14 ± 0.02	3.44 ± 0.25	7.66 ± 0.74
	15	3.04 ± 0.52	6.26 ± 0.50	2.75 ± 0.50	3.11 ± 0.47	5.20 ± 1.19
	30	3.64 ± 0.14	4.17 ± 0.07	9.94 ± 0.62	7.36 ± 1.23	4.02 ± 0.64
Methyl butyrate	1	0.07 ± 0.00	37.79 ± 5.81	1.25 ± 0.05	9.57 ± 2.40	59.17 ± 2.74
	15	31.16 ± 0.93	45.19 ± 4.63	6.28 ± 1.34	11.28 ± 6.34	52.58 ± 1.05
	30	40.28 ± 1.98	37.58 ± 7.55	55.23 ± 3.41	55.23 ± 3.41	32.49 ± 0.31
Sulfur compounds						
Diallyl sulfide	1	N.D.	58.78 ± 3.39	1.02 ± 0.03	16.49 ± 1.28	68.64 ± 0.71
	15	N.D.	48.82 ± 7.43	24.09 ± 1.49	18.70 ± 1.29	57.79 ± 1.49
	30	N.D.	77.83 ± 6.51	336.25 ± 7.74	75.05 ± 0.31	46.92 ± 0.71
Diallyl disulfide	1	N.D.	637.52 ± 91.24	15.25 ± 1.49	310.47 ± 5.98	792.05 ± 18.91
	15	N.D.	1032.52 ± 13.76	471.67 ± 18.62	364.88 ± 1.31	1263.85 ± 82.25
	30	N.D.	1028.04 ± 16.54	7727.74 ± 25.22	2007.85 ± 1.66	1074.87 ± 39.62
Dimethyl trisulfide	1	N.D.	17.71 ± 0.45	N.D.	28.60 ± 1.42	15.95 ± 3.41
	15	N.D.	15.88 ± 0.26	N.D.	34.64 ± 0.52	22.02 ± 3.54
	30	N.D.	2.56 ± 0.12	N.D.	57.09 ± 1.23	1.38 ± 0.03
Allyl methyl disulfide	1	N.D.	209.91 ± 18.49	2.09 ± 0.12	67.84 ± 2.77	220.43 ± 7.76
	15	N.D.	174.95 ± 6.75	34.92 ± 0.36	59.44 ± 0.77	231.33 ± 2.37
	30	N.D.	414.90 ± 4.36	934.66 ± 8.02	776.57 ± 0.61	235.75 ± 4.96

*Note:* ^a^All volatile components are given in Table S4–S8.

Abbreviations: N.D., Not detected; SE, standard error.

Yogurt is typically characterized characteristic taste of sharp acid and a green apple flavor (Tamime and Robinson 2000). Acidity is a crucial factor for the taste acceptance of yogurt (Cheng 2010). Eight carboxylic acids were detected in the yogurt samples. The carboxylic acids identified in the yogurt samples were found in higher amounts compared to other volatile compounds (excluding sulfur compounds). Octanoic acid, acetic acid, butyric acid, decanoic acid, and benzoic acid were detected in all samples throughout the storage period. Valeric acid was only detected in the control sample. Acetic acid, one of the key acids in yogurt, was found in higher amounts in yogurt with added garlic compared to the control sample. Various studies have found acetic acid, octanoic acid, and hexanoic acid in garlic. Acetic acid is associated with the characteristic sour taste of yogurt, while butyric acid is linked to the taste of butter. Hexanoic acid, on the other hand, is associated with a sour, fruity taste in yogurt (Dan et al. 2017). Among the yogurt samples with added garlic, Sample D (added Taşköprü garlic) showed higher amounts of acidic components compared to the others. Similarly, in a study conducted by Arslaner (2020), the amounts of octanoic acid, acetic acid, and butyric acid were found to be higher in yogurt with added garlic compared to the control sample.

Six aldehydes and six ketones were identified in the yogurt samples. In this study, a total of 6 aldehydes, mostly hexenal and acetone, were detected in yogurt samples. Acetaldehyde is one of the primary aroma components of yogurt. Many studies indicate that the amount of acetaldehyde decreases during the storage of yogurt. In the present study, acetaldehyde was detected in all samples on the first day of storage, but by the end of storage, it could not be detected in any of the samples. Benzaldehyde which contributes to the almond‐like aromatic flavor in fermented products (Albert et al. 2023), was detected in all samples during the storage period. Hexanal is one of the important aldehydes that contribute to the grass‐like flavor of yogurt (Albert et al. 2023). Hexanal and acetone were detected in all samples throughout the storage period. Additionally, nonanal and 2‐Butenal 3‐methyl‐ were determined at different storage periods in yogurt samples, and they had fluctuating levels. Acetone and 2‐butanone have similar aroma characteristics and have a significant impact on the flavor of yogurt. Both components contribute positively to the sweet‐fruity taste of yogurt (Albert et al. 2023).

A total of 6 ketones, mainly diacetyl acetoin and 2‐nonanon, were detected in yogurt samples. These components were present in higher quantities than others. The unique taste of yogurt occurs through the action of various C4 molecules in fermented dairy products, such as diacetyl, and acetoin. Among these, diacetyl is important due to its low perception threshold (Chen et al. 2017). In this study, diacetyl was detected in all samples during storage except the day 30. Acetoin is the reduced form of diacetyl, and its taste is significantly weaker than that of diacetyl. However, both of these compounds' characteristics of flavor are similar, and they contribute to “buttery” aroma of yogurt (Cheng 2010). Acetoin was detected in all samples throughout the storage period. Acetyl propionyl was detected in all samples except for the control sample.

In this study, a total of 4 alcohol components were identified. Among these, 2‐heptanol and phenethyl alcohol were not detected in the control sample. 2‐pentanol was detected in all samples except Sample C (yogurt with Maraş garlic), with fluctuating levels on different storage days. 1‐hexanol was only detected in all samples during the 30 days of storage.

Esters are found in low concentrations in dairy products, they are volatile compounds that are mostly found in dairy products and contribute to the flavor of yogurt (Cheng 2010; Puniya 2016). In the study, 5 ester components were identified. Among these, isobutyl isobutyrate, butyl butyrate, methyl butyrate, and butyl butyrate were detected in all samples, while isobutyl butyrate was detected in all samples except Sample B (yogurt with Mersin garlic). Among the esters, methyl butyrate was found in higher amounts compared to the other ester components.

Garlic primarily derives its characteristic aroma from thiosulfinates like allicin and its degradation products such as diallyl disulfide and diallyl trisulfide (Molina‐Calle et al. 2017). Allicin, resulting from mechanical processes like cutting or crushing garlic, readily breaks down into volatile organosulfur compounds such as diallyl sulfide, diallyl disulfide, diallyl trisulfide, and allyl methyl disulfide, which are precursors to garlic's pungent odor (Abe et al. 2020). These components not only provide garlic with its characteristic smell and aroma but also possess properties such as antimicrobial, antioxidant, anti‐inflammatory, anti‐thrombotic, anti‐atherosclerotic, anti‐hyperlipidemic, and pro‐circulatory effects (Scheffler et al. 2019; Sharma et al. 2024). Sulfur compounds identified in yogurt samples constituted most of the volatile compound profile. A total of 14 sulfur compounds were found. As all identified sulfur compounds originated from garlic, as expected, no sulfur compound was found in the control sample. In contrast to this study, Arslaner (2020) found allyl methyl disulfide (1‐Propene. 3‐(methylthio)) compound in the control sample. Among the sulfur compounds identified in yogurt samples, Diallyl disulfide was the most abundant compound in terms of quantity. Studies conducted on garlic grown in different regions of Turkey—the same garlic used in this study—indicate that diallyl disulfide is the major aroma component of garlic. The sulfur compounds detected in this study were found at the highest levels in Sample A (yogurt with Ankara garlic) and D (yogurt with Taşköprü garlic) on days 1 and 15 of storage.

### 3.7 Sensory Properties

**FIGURE 5:**
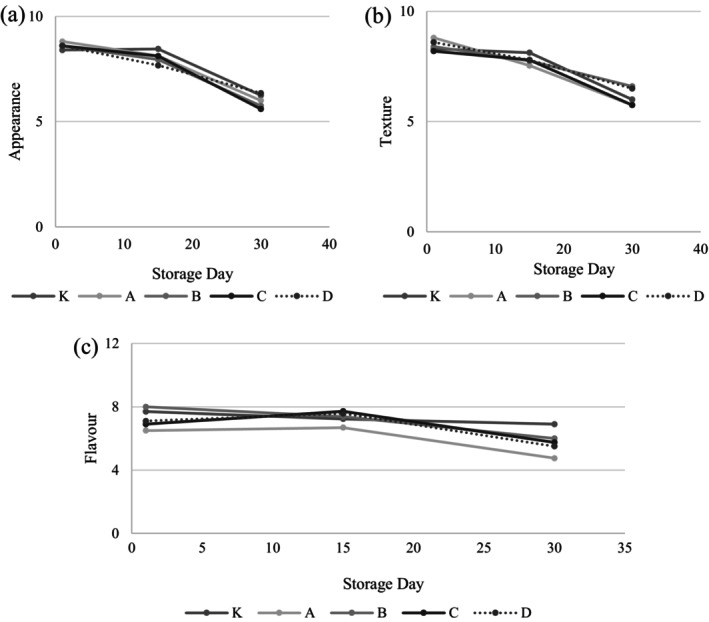
Sensorial attributes of yogurt samples. K: Control sample (not containing garlic), A: Yogurt containing Ankara garlic, B: Yogurt containing Mersin garlic C: Yogurt containing Maraş garlic, D: Yogurt containing Taşköprü garlic.

The sensory evaluation of yogurt samples, focusing on appearance, texture, and flavor, is present in Figure 5 and Table S9. This evaluation revealed that the inclusion of garlic in the yogurt samples did not alter their appearance or textural properties. Specifically, no variations were observed in hardness, cohesiveness, or the index of viscosity, except for a non‐significant change in consistency as perceived by the panelists regarding the samples' structural properties. However, the flavor profiles of the samples exhibited significant differences (*p* < 0.05), primarily due to the varied volatile component profiles stemming from garlic of different genotypes and ecotypes. Yogurt samples with Ankara garlic (A coded sample), known for its high volatile content, received notably lower flavor scores from the panelists compared to the control sample in all storage days, attributed to its strong and pronounced taste (*p* < 0.05). Conversely, yogurt samples containing Mersin garlic (B‐coded sample) were favored over the control sample after 15 days of storage. By the day 15, samples coded C and D also achieved higher scores than the control for their aroma qualities. As a result of the volatile component analysis of these samples, it is seen that the components of diallyl sulfide, diallyl disulfide, and diallyl trisulfide, which are responsible for the sharp taste and smell of garlic, were determined in lower amounts than other samples on the 15th day. Therefore, the intense garlic taste in samples B and C may have been felt less than the other samples and may have caused the flavor of samples B and C to be perceived positively. Nevertheless, storage duration significantly impacted all sensory parameters (*p* < 0.05).

By the 30th day, there was a marked decrease in sensory scores across all parameters, with the decline in flavor being particularly pronounced in yogurt samples containing garlic. Research by Akan et al. (2022), Gündoğdu, Cakmakci, and Dagdemir (2009), and Arslaner (2020) on the sensory impact of garlic in yogurt production corroborated these findings, indicating that garlic yogurt samples peaked in sensory appeal at the onset of storage, with scores diminishing over time. The panelists negatively assessed the changes in flavor and texture that occurred with prolonged storage.

## 4 Conclusion

Garlic, which is a very important plant in terms of its positive effects on health, has been used in the production of yogurt, which also has many health benefits, and a functional product has been obtained. As the region where garlic grows differs, the functional properties of the products also change. In this study, where the effect of garlic with different genotypic characteristics on yogurt production was investigated, it was observed that the differences in garlic were similarly reflected in the product to which it was added. The nutritional value and sensory properties of yogurts improved with the addition of garlic. However, the addition of garlic did not affect the composition and physical properties of the yogurts. The addition of garlic negatively affected the growth of the starter culture used in yogurt production during storage due to its antimicrobial effect. Depending on the garlic genotype, which has a sharp taste and smell (especially A‐coded garlic), it may also cause a taste in yogurt that is not liked by the panelists. While the flavor scores were higher in sample B on the 1st day of storage because the amounts of volatile compounds were lower, this had the opposite effect as storage progressed. In samples A and D, a sharp bitter taste occurred in the yogurts due to the presence of intense sulfur components, so the taste scores of the samples were lower than the other samples. The highest antioxidant, phenolic, and volatile components were detected in yogurt samples added to Taşköprü garlic, which is geographically registered in Turkey. In addition, the amount of volatile sulfur component was found to be higher in the sample with Taşköprü garlic addition compared to the others.

## Author Contributions

H. Ceren Akal and Gökçe Eminoğlu: Conceptualization; data curation; investigation; methodology; supervision; visualization; writing – original draft; writing – review and editing. Selen Akan: Conceptualization; methodology; resources.

## Conflicts of Interest

The authors declare no conflicts of interest.

## Supporting information


Data S1.

## Data Availability

The data that support the findings of this study are available on request from the corresponding author. The data are not publicly available due to privacy or ethical restrictions.
