# Supplementary material for: The Influence of Different Garlic Genotypes on Yogurt Production
Source: Food Sci Nutr. 2025 Mar 4;13(3):e4606. doi: 10.1002/fsn3.4606 (PMC11880621; doi:10.1002/fsn3.4606)
Supplement: Supplementary file 1 — Data S1. [file FSN3-13-e4606-s001.docx]

Table S1. Antioxidant activity (AA) and Total phenolics content (TPC) on yogurt samples(mean ± SE)

| Samples | Days | AA (%) | TPC (mg GAE/ 100g) |
| --- | --- | --- | --- |
| K | 1 | 32.82±1.55^Ba^ | 21±0.09^Ba^ |
|  | 15 | 29.62±2.69^b^ | 33±0.00 ^a^ |
|  | 30 | 27.98±4.80^c^ | 10±0.04 ^b^ |
| A | 1 | 54.95±4.80^Aa^ | 26±0.01^Ba^ |
|  | 15 | 35.26±0.13^b^ | 34±0.05^a^ |
|  | 30 | 24.83±0.66^c^ | 9±0.02^b^ |
| B | 1 | 62.07±6.35^Aa^ | 24±0.01^Ba^ |
|  | 15 | 45.51±9.62^b^ | 38±0.13^a^ |
|  | 30 | 27.32±5.13^c^ | 9±0.00^b^ |
| C | 1 | 51.70±7.12^Aa^ | 30±0.02^ABa^ |
|  | 15 | 45.64±1.03^b^ | 38±0.08^a^ |
|  | 30 | 21.36±8.44^c^ | 8±0.01^b^ |
| D | 1 | 64.86±2.63^Aa^ | 61±0.13^Aa^ |
|  | 15 | 46.28±1.92^b^ | 41±0.06^a^ |
|  | 30 | 40.56±9.11^c^ | 15±0.06^b^ |

K: Control sample (not containing garlic), A: Yogurt containing Ankara garlic, B: Yogurt containing Mersin garlic C: Yogurt containing Maraş garlic, D: Yogurt containing Taşköprü garlic The different lowercase letters in the same column indicate significant differences during storage period (P<0.05). The different uppercase letters in the same column indicate significant differences among the samples (P<0.05) SE:Standard error

Table S2. Microbiological properties on yogurt samples (log cfu/g) (mean ± SE)

| Samples | Days | TMAB | *Streptococcus* ssp | *Lactobacillus* ssp. |
| --- | --- | --- | --- | --- |
| K | 1 | 8.23±0.01^AB^ | 7.25±0.02^ABC^ | 6.25±0.02^A^ |
|  | 15 | 8.58±0.05^A^ | 8.21±0.21^A^ | 6.22±0.01^A^ |
|  | 30 | 8.36±0.00^AB^ | 7.75±0.05^AB^ | 5.66±0.00^B^ |
| A | 1 | 8.42±0.01^AB^ | 7.36±0.04 ^ABC^ | 5.89±0.14^AB^ |
|  | 15 | 7.39±0.05^BCD^ | 7.43±0.39 ^ABC^ | 4.15±0.15^D^ |
|  | 30 | 8.38±0.02 ^AB^ | 7.69±0.27^AB^ | 3.66±0.14 ^D^ |
| B | 1 | 8.16±0.03 ^AB^ | 6.87±0.55^BC^ | 5.97±0.15 ^AB^ |
|  | 15 | 7.91±0.06^ABC^ | 8.26±0.23^A^ | 5.15±0.11^C^ |
|  | 30 | 7.33±0.13^CD^ | 7.35±0.01 ^ABC^ | 4.04±0.09 ^D^ |
| C | 1 | 8.31±0.02 ^AB^ | 6.83±0.02^BC^ | 6.04±0.01 ^AB^ |
|  | 15 | 7.78±0.12 ^ABC^ | 8.21±0.19^A^ | 5.15±0.03 ^C^ |
|  | 30 | 6.48±0.01^D^ | 7.10±0.08 ^ABC^ | 4.11±0.06 ^D^ |
| D | 1 | 8.25±0.02 ^AB^ | 7.49±0.03 ^ABC^ | 5.79±0.04 ^AB^ |
|  | 15 | 7.84±0.10 ^ABC^ | 8.42±0.05 ^ABC^ | 4.72±0.12 ^C^ |
|  | 30 | 7.08±0.68^CD^ | 7.59±0.40^AB^ | 3.95±0.03 ^D^ |

K: Control sample (not containing garlic), A: Yogurt containing Ankara garlic, B: Yogurt containing Mersin garlic C: Yogurt containing Maraş garlic, D: Yogurt containing Taşköprü garlic. The different uppercase letters in the same column indicate significant differences among the samples (P<0.05) SE:Standard error

Table S3. Textural properties and whc results of yogurt samples(mean ± SE)

| Samples | Days | Firmness g | Consistency g.sec | Cohesiveness g | Index of Viscosity g.sec | WHC |
| --- | --- | --- | --- | --- | --- | --- |
| K | 1 | 69.79±4.39^c^ | 1210.13±75.97^Ab^ | 56.53±4.65 | 109.66±6.59 | 38.44±0.49^b^ |
|  | 15 | 91.60±3.64^b^ | 1468.79±84.49^b^ | 73.26±6.66 | 133.67±9.29 | 48.92±0.47^a^ |
|  | 30 | 103.5±0.56^a^ | 1666.64±32.75^a^ | 75.74±4.40 | 92.96±5.43 | 44.90±1.60^a^ |
| A | 1 | 62.72±5.42 | 1064.00±70.95^B^ | 53.06±5.05 | 104.32±9.57 | 40.71±0.50 |
|  | 15 | 69.61±6.88 | 1116.20±91.33 | 58.37±4.92 | 110.16±17.40 | 45.95±1.17 |
|  | 30 | 83.22±9.16 | 1330.14±89.39 | 70.32±5.08 | 135.02±7.65 | 43.60±2.73 |
| B | 1 | 61.12±3.30 | 1042.94±49.33^B^ | 50.74±3.33 | 99.29±6.14 | 39.45±1.69 |
|  | 15 | 68.01±1.79 | 1144.16±26.57 | 57.91±1.49 | 112.84±4.09 | 46.26±1.75 |
|  | 30 | 79.58±3.82 | 1270.37±67.15 | 65.18±3.62 | 69.47±46.03 | 42.11±0.11 |
| C | 1 | 59.86±4.41 | 1029.22±72.59^B^ | 49.99±4.73 | 98.17±7.95 | 39.77±0.05 |
|  | 15 | 65.03±3.03 | 1098.32±60.03 | 54.04±3.80 | 105.44±7.68 | 45.58±0.38 |
|  | 30 | 69.35±3.65 | 1179.26±88.42 | 58.34±4.57 | 114.59±7.52 | 42.83±1.48 |
| D | 1 | 58.94±4.32 | 999.57±75.98 ^B^ | 47.98±3.88 | 94.14±5.93 | 40.85±2.25 |
|  | 15 | 68.50±4.41 | 1122.45±68.62 | 56.48±5.01 | 107.25±8.13 | 43.02±5.80 |
|  | 30 | 83.62±5.14 | 1322.10±94.03 | 65.68±2.18 | 76.11±7.76 | 45.98±2.02 |

K: Control sample (not containing garlic), A: Yogurt containing Ankara garlic, B: Yogurt containing Mersin garlic C: Yogurt containing Maraş garlic, D: Yogurt containing Taşköprü garlic The different lowercase letters in the same column indicate significant differences during storage period (P<0.05). The different uppercase letters in the same column indicate significant differences among the samples (P<0.05) SE:Standard error

Table S4. Carboxylic acids in yogurt samples during 30 days of storage (µg/100 g) (mean ± SE)

| **Acids** | **Days** | **K** | **A** | **B** | **C** | **D** |
| --- | --- | --- | --- | --- | --- | --- |
| Octanoic acid | 1 | 1.38±0.04 | 57.94±2.50 | 3.34±0.50 | 93.12±2.53 | 120.72±5.65 |
|  | 15 | 72.55±1.22 | 53.89±2.30 | 83.72±0.56 | 87.59±5.06 | 103.07±8.84 |
|  | 30 | 24.26±3.23 | 52.04±0.18 | 159.67±2.32 | 58.67±0.12 | 33.43± 0.84 |
| Hexanoic acid | 1 | N.D. | 141.44±13.15 | 5.45±0.53 | 124.42±3.30 | 251.57±7.70 |
|  | 15 | N.D. | 165.16±8.79 | 114.31±2.50 | 137.32±1.64 | 242.91±3.58 |
|  | 30 | 23.53±2.49 | N.D. | 163.25±2.08 | 35.84±0.21 | 34.11±2.88 |
| Acetic acid | 1 | 2.52±0.32 | 81.90±5.14 | 3.24±0.71 | 87.58±2.06 | 137.09±0.86 |
|  | 15 | 112.52±4.85 | 114.94±8.91 | 53.78±1.26 | 55.11±1.83 | 147.37±10.35 |
|  | 30 | 44.23±0.99 | 38.58±3.35 | 93.99±1.98 | 58.20±0.63 | 37.45±0.64 |
| Butyric acid | 1 | 1.72±0.06 | 73.72±14.24 | 1.79±0.25 | 35.83±0.86 | 72.17±4.37 |
|  | 15 | 54.83±4.29 | 58.24±3.40 | 28.52±1.32 | 27.57±0.80 | 73.47±8.26 |
|  | 30 | 23.23±2.28 | 23.25±3.40 | 95.96±1.91 | 32.28±0.81 | 20.16±1.74 |
| Valeric acid (pentanoic acid) | 1 | 0.04±0.01 | N.D. | N.D. | N.D. | N.D. |
|  | 15 | 2.67±0.49 | N.D. | N.D. | N.D. | N.D. |
|  | 30 | 5.94±1.45 | N.D. | N.D. | N.D. | N.D. |
| Nonanoic acid | 1 | 0.19±0.16 | 2.74±0.99 | 0.10±0.02 | 2.47±0.25 | 6.35±1.14 |
|  | 15 | N.D. | N.D. | N.D. | 1.34±0.77 | N.D. |
|  | 30 | N.D. | N.D. | 20.79±1.77 | N.D. | N.D. |
| Decanoic acid | 1 | 0.36±0.11 | 51.63±16.79 | 1.33±0.30 | 55.95±1.55 | 99.26±3.47 |
|  | 15 | 44.32±1.94 | 75.82±5.19 | 31.11±0.17 | 39.96±11.24 | 53.89±4.24 |
|  | 30 | 34.46±1.46 | 36.02±5.31 | 204.92±1.00 | 40.10±0.64 | 32.65±1.59 |
| Benzoic acid | 1 | 0.05±0.02 | 6.47±1.85 | 0.24±0.01 | 23.27±0.49 | 22.10±6.57 |
|  | 15 | 5.43±0.26 | 12.89±1.49 | 5.03±0.59 | 12.23±7.63 | 11.72±8.26 |
|  | 30 | 16.84±1.13 | 3.82±0.57 | 15.34±0.63 | 3.41±0.17 | 4.32±0.46 |

K: Control sample (not containing garlic), A: Yogurt containing Ankara garlic, B: Yogurt containing Mersin garlic C: Yogurt containing Maraş garlic, D: Yogurt containing Taşköprü garlic N.D: Not detected, SE:Standard error

Table S5. Aldehydes and ketones in yogurt samples during 30 days of storage (µg/100 g) (mean ± SE)

| **Aldehydes** | Days | K | A | B | C | D |
| --- | --- | --- | --- | --- | --- | --- |
| Acetaldehyde | 1 | 0.26±0.03 | 1.75±0.08 | 0.71±0.04 | 1.55±0.40 | 1.52±0.38 |
|  | 15 | N.D. | N.D. | N.D. | N.D. | N.D. |
|  | 30 | N.D. | N.D. | N.D. | N.D. | N.D. |
| Benzaldehyde | 1 | 0.22±0.01 | 5.35±0,32 | 0.19±0.00 | 6.47±1.12 | 11.95±0.55 |
|  | 15 | 4.88±0.47 | 11.36±4.16 | 7.01±0.13 | 12.65±3.26 | 14.79±0.10 |
|  | 30 | 2.48±0.16 | 10.13±6.75 | 0.75±0.61 | 8.44±0.34 | 7.55±0.12 |
| Nonanal | 1 | N.D. | N.D. | 0.06±0.02 | 1.31±0.02 | 12.97±9.05 |
|  | 15 | 2.16±0.07 | 4.84±0.23 | 3.13±0.86 | 3.13±1.32 | 6.27±0.96 |
|  | 30 | N.D. | N.D. | N.D. | N.D. | N.D. |
| Hexenal | 1 | 0.35±0.12 | 12.55±1.05 | 0.36±0.04 | 5.78±0.26 | 15.17±1.26 |
|  | 15 | 7.36±0.43 | 20.03±1.44 | 10.12±2.30 | 6.19±0.16 | 22.48±2.96 |
|  | 30 | 4.02±0.79 | 5.91±0.34 | 3.60±0.48 | 6.66±1.06 | 5.86±0.51 |
| Acetone (2-Propanone) | 1 | 0.48±0.04 | 27.02±2.12 | 1.43±0.03 | 22.52±1.67 | 78.97±11.52 |
|  | 15 | 2.24±0.05 | 29.01±3.04 | 8.69±0.3 | 14.54±1.44 | 35.45±4.05 |
|  | 30 | 5.99±0.16 | 10.45±0.61 | 9.00±0.26 | 13.75±2.16 | 16.86±0.06 |
| 2-Butenal 3-methyl- | 1 | N.D. | N.D. | 0.27±0.02 | 3.39±0.00 | 10.09±1.45 |
|  | 15 | 7.41±0.67 | 6.54±0.10 | 3.45±0.14 | N.D. | N.D. |
|  | 30 | 3.41±0.08 | N.D. | N.D. | 2.92±0.58 | 3.03±0.01 |
| **Ketons** | Days | K | A | B | C | D |
| 2-Butanone | 1 | 0.33± 0.22 | N.D. | 0.42±0.10 | N.D. | 4.82±0.50 |
|  | 15 | 0.30±0.03 | 6.47±1.20 | 7.66±0.51 | 6.95±0.20 | N.D. |
|  | 30 | 1.55±0.53 | 4.45±1.28 | 12.56±0.36 | N.D. | 4.63±0.55 |
| 2.3-Butanedione (diacetyl) | 1 | 0.58±0.04 | 11.05±1.99 | 0.50±0.08 | 9.35±0.36 | 16.40±2.13 |
|  | 15 | 8.72±0.27 | 7.65±1.85 | 5.06±0.40 | 7.83±0.15 | 10.82±0.35 |
|  | 30 | N.D. | N.D. | N.D. | N.D. | N.D. |
| 2-Nonanon | 1 | 0.53±0.31 | 15.31±2.86 | 0.36±0.06 | 7.00±0.60 | 17.56±0.82 |
|  | 15 | 22.93±1.53 | 16.40±1.60 | 8.71±0.37 | 6.82±0.27 | 19.85±2.70 |
|  | 30 | 145.57±2.20 | N.D. | 156.88±1.24 | N.D. | 90.72±0.39 |
| 2-Undecanone | 1 | N.D. | N.D. | N.D. | N.D. | N.D. |
|  | 15 | 1.79±0.36 | N.D. | 1.74±0.14 | N.D. | N.D. |
|  | 30 | 3.88±0.52 | N.D. | 9.54±0.19 | N.D. | 3.18±0.50 |
| 2-Butanone. 3-hydroxy- (Acetoin) | 1 | 2.15±0.02 | 68.85±2.52 | 1.80±0.14 | 42.63±0.98 | 75.32±1.43 |
|  | 15 | 52.50±1.34 | 63.99±3.55 | 25.32±0.76 | 42.58±3.32 | 77.68±2.31 |
|  | 30 | 16.44±1.69 | 25.94±0.17 | 89.50±1.27 | 28.84±0.28 | 22.88±0.68 |
| Acetyl propionyl (2.3-Pentanedione) | 1 | N.D. | 10.49±1.84 | 0.37±0.03 | 5.37±0.28 | 11.04±1.11 |
|  | 15 | N.D. | 9.21±0.09 | 3.91±0.07 | N.D. | 9.27±0.22 |
|  | 30 | N.D. | N.D. | N.D. | N.D. | N.D. |

K: Control sample (not containing garlic), A: Yogurt containing Ankara garlic, B: Yogurt containing Mersin garlic C: Yogurt containing Maraş garlic, D: Yogurt containing Taşköprü garlic N.D: Not detected,SE:Standard error

Table S6. Alcohols in yogurt samples during 30 days of storage (µg/100 g) (mean ± SE)

| **Alcohols** | Days | K | A | B | C | D |
| --- | --- | --- | --- | --- | --- | --- |
| 2-Heptanol | 1 | N.D. | N.D. | N.D. | N.D. | N.D. |
|  | 15 | N.D. | N.D. | N.D. | N.D. | N.D. |
|  | 30 | N.D. | 5.38±1.06 | 5.41±0.58 | 3.56±0.14 | 2.30±0.02 |
| Phenethyl alcohol (Benzeneethanol | 1 | N.D. | N.D. | N.D. | N.D. | N.D. |
|  | 15 | N.D. | N.D. | N.D. | N.D. | N.D. |
|  | 30 | N.D. | 6.56±0.25 | 65.10±3.70 | 2.56±0.24 | 5.55±0.52 |
| 2-Pentanol | 1 | N.D. | N.D. | N.D. | N.D. | 8.44±0.18 |
|  | 15 | 1.48±0.10 | 0.97±0.97 | N.D. | N.D. | N.D. |
|  | 30 | 1.56±0.24 | N.D. | 22.65±1.00 | N.D. | N.D. |
| 1-Hexanol | 1 | N.D. | N.D. | N.D. | N.D. | N.D. |
|  | 15 | N.D. | N.D. | N.D. | N.D. | N.D. |
|  | 30 | 2.58±0.61 | 5.70±0.62 | 6.91±0.18 | 4.83±0.66 | 7.85±0..56 |

K: Control sample (not containing garlic), A: Yogurt containing Ankara garlic, B: Yogurt containing Mersin garlic C: Yogurt containing Maraş garlic, D: Yogurt containing Taşköprü garlic N.D: Not detected ,SE:Standard error

Table S7. Esters in yogurt samples during 30 days of storage (µg/100 g) (mean ± SE)

| **Esters** | Days | K | A | B | C | D |
| --- | --- | --- | --- | --- | --- | --- |
| Isobutyl isobutyrate | 1 | 0.37±0.17 | 7.94±0.95 | 0.40±0.13 | 1.52±0.21 | 6.26±0.72 |
|  | 15 | 5.80±0.50 | 4.05±0.55 | 0.92±0.04 | 2.48±0.40 | 8.13±0.50 |
|  | 30 | 5.11±8.87 | 5.03±0.46 | 7.21±0.67 | 6.67±0.44 | 4.84±0.41 |
| Butyl butyrate | 1 | 0.57±0.15 | 3.47±0.23 | 0.14±0.02 | 3.44±0.25 | 7.66±0.74 |
|  | 15 | 3.04±0.52 | 6.26±0.50 | 2.75±0.50 | 3.11±0.47 | 5.20±1.19 |
|  | 30 | 3.64±0.14 | 4.17±0.07 | 9.94±0.62 | 7.36±1.23 | 4.02±0.64 |
| Methyl butyrate | 1 | 0.07±0.00 | 37.79±5.81 | 1.25±0.05 | 9.57±2.40 | 59.17±2.74 |
|  | 15 | 31.16±0.93 | 45.19±4.63 | 6.28±1.34 | 11.28±6.34 | 52.58±1.05 |
|  | 30 | 40.28±1.98 | 37.58±7.55 | 55.23±3.41 | 55.23±3.41 | 32.49±0.31 |
| Isobutyl butyrate | 1 | N.D. | 5.66±0.69 | N.D. | 1.44±0.16 | 5.46±0.25 |
|  | 15 | 2.57±0.03 | 3.77±0.88 | N.D. | 1.56±0.21 | 5.45±1.47 |
|  | 30 | 6.15±0.44 | 4.57±0.58 | N.D. | 8.91±0.44 | N.D. |
| Butyl butyrate | 1 | 0.06±0.01 | 6.17±0.32 | 0.14±0.02 | 3.44±0.25 | 7.66±0.74 |
|  | 15 | 3.51±0.05 | 2.57±0.24 | 2.62±0.37 | 3.11±0.47 | 5.20±1.19 |
|  | 30 | 3.64±0.14 | 4.17±0.07 | 2.77±0.44 | 7.36±1.23 | 4.02±0.64 |

K: Control sample (not containing garlic), A: Yogurt containing Ankara garlic, B: Yogurt containing Mersin garlic C: Yogurt containing Maraş garlic, D: Yogurt containing Taşköprü garlic N.D: Not detected ,SE:Standard error

Table S8. Sulphur compunds in yogurt samples during 30 days of storage (µg/100 g) (mean ± SE)

| **Sulphur compunds** | Days | K | A | B | C | D |
| --- | --- | --- | --- | --- | --- | --- |
| 1-Propene. 3-(methylthio)- | 1 | N.D. | 22.77±4.68 | 0.25±0.06 | 8.73±0.30 | 24.90±0.61 |
|  | 15 | N.D. | 25.17±1.57 | 3.48±0.16 | 8.94±0.00 | 30.31±2.58 |
|  | 30 | N.D. | 55.11±1.49 | 144.35±0.45 | 76.59±1.56 | 37.58±1.42 |
| Disulfide. dimethyl | 1 | N.D. | 15.87±2.44 | 0.09±0.04 | 5.92±0.46 | 20.28±3.00 |
|  | 15 | N.D. | 10.42±0.75 | 1.39±0.13 | 3.75±0.16 | 16.03±1.06 |
|  | 30 | N.D. | 10.90±0.21 | 4.95±0.35 | 43.96±1.70 | 6.91±1.38 |
| Diallyl sulfide | 1 | N.D. | 58.78±3.39 | 1.02±0.03 | 16.49±1.28 | 68.64±0.71 |
|  | 15 | N.D. | 48.82±7.43 | 24.09±1.49 | 18.70±1.29 | 57.79±1.49 |
|  | 30 | N.D. | 77.83±6.51 | 336.25±7.74 | 75.05±0.31 | 46.92±0.71 |
| Allyl methyl disulfide | 1 | N.D. | 209.91±18.49 | 2.09±0.12 | 67.84±2.77 | 220.43±7.76 |
|  | 15 | N.D. | 174.95±6.75 | 34.92±0.36 | 59.44±0.77 | 231.33±2.37 |
|  | 30 | N.D. | 414.90±4.36 | 934.66±8.02 | 776.57±0.61 | 235.75±4.96 |
| Dimethyl trisulfide | 1 | N.D. | 17.71±0.45 | N.D. | 28.60±1.42 | 15.95±3.41 |
|  | 15 | N.D. | 15.88±0.26 | N.D. | 34.64±0.52 | 22.02±3.54 |
|  | 30 | N.D. | 2.56±0.12 | N.D. | 57.09±1.23 | 1.38±0.03 |
| 1-Allyl-2-isopropyldisulfane | 1 | N.D. | 3.31±0.42 | N.D. | N.D. | N.D. |
|  | 15 | N.D. | 8.56±0.51 | N.D. | N.D. | N.D. |
|  | 30 | N.D. | 10.98±0.46 | 13.85±1.98 | 8.86±0.15 | 6.10±0.86 |
| (Z)-1-Allyl-2-(prop-1-en-1-yl)disulfane | 1 | N.D. | 33.38±0.39 | 0.90±0.24 | 27.22±2.01 | 35.66±1.49 |
|  | 15 | N.D. | 57.34±1.18 | 35.16±0.19 | 38.99±0.17 | 69.57±1.60 |
|  | 30 | N.D. | 56.39±7.06 | 164.82±4.71 | 127.00±0.88 | 27.75±0.02 |
| Diallyl disulphide | 1 | N.D. | 637.52±91.24 | 15.25±1.49 | 310.47±5.98 | 792.05±18.91 |
|  | 15 | N.D. | 1032.52±13.76 | 471.67±18.62 | 364.88±1.31 | 1263.85±82.25 |
|  | 30 | N.D. | 1028.04±16.54 | 7727.74±25.22 | 2007.85±1.66 | 1074.87±39.62 |
| 3H-1.2-Dithiole | 1 | N.D. | 36.11±8.15 | 0.54±0.04 | 24.54±1.41 | 53.67±2.49 |
|  | 15 | N.D. | 57.81±8.86 | 20.05±0.50 | 27.64±1.85 | 67.55±18.16 |
|  | 30 | N.D. | 24.16±1.38 | 28.89±5.08 | 37.40±2.09 | 13.24±3.17 |
| Trisulfide. methyl 2-propenyl | 1 | N.D. | 148.03±6.65 | 0.86±0.09 | 155.89±0.76 | 194.25±15.76 |
|  | 15 | N.D. | 263.91±8.54 | 30.20±0.15 | 272.99±20.72 | 329.22±14.97 |
|  | 30 | N.D. | 85.31±2.02 | 38.52±1.43 | 391.85±5.03 | 133.55±1.16 |
| Trisulfide. di-2-propenyl | 1 | N.D. | 88.73±13.64 | 1.14±0.22 | N.D. | 163.31±4.03 |
|  | 15 | N.D. | 305.83±2.98 | 56.93±1.78 | 213.33±0.66 | 332.45±2.41 |
|  | 30 | N.D. | 168.38±28.51 | 141.26±5.08 | 208.17±0.58 | 47.73±1.50 |
| 3-Vinyl-1.2-dithiacyclohex-4-ene | 1 | N.D. | 253.35±26.04 | 3.54±0.62 | 137.68±1.45 | 344.93±27.27 |
|  | 15 | N.D. | 305.42±37.31 | 98.93±3.32 | 159.25±0.29 | 333.12±42.81 |
|  | 30 | N.D. | 93.86±21.06 | 111.68±5.23 | 127.23±0.27 | N.D. |
| (Z)-1-Allyl-3-(prop-1-en-1-yl)trisulfane | 1 | N.D. | N.D. | 0.13±0.01 | 8.50± | 1.13±0.00 |
|  | 15 | N.D. | 16.85±0.25 | N.D. | 11.80± | 0.26±17.24 |
|  | 30 | N.D. | N.D. | N.D. | 8.76± | 0.47±0.00 |
| Thiophene. 3.4-dimethyl- | 1 | N.D. | N.D. | 0.07±0.00 | 7.04±0.07 | 3.39±0.91 |
|  | 15 | N.D. | 5.21±3.05 | N.D. | 4.43±2.78 | 8.91±0.84 |
|  | 30 | N.D. | 1.83±0.05 | 7.75±0.18 | 3.98±0.92 | 2.22±0.21 |

K: Control sample (not containing garlic), A: Yogurt containing Ankara garlic, B: Yogurt containing Mersin garlic C: Yogurt containing Maraş garlic, D: Yogurt containing Taşköprü garlic N.D: Not detected ,SE:Standard error

Table S9. Sensorial scores of yogurt samples during 30 days of storage (mean ± SE)

| Samples | Days | Appearance | Texture | Flavour |
| --- | --- | --- | --- | --- |
| K | 1 | 8.40±0.00^a^ | 8.30±0.10^a^ | 7.70±0.30^Aa^ |
|  | 15 | 8.45±0.12^b^ | 8.13±0.01^b^ | 7.24±0.09^a^ |
|  | 30 | 6.25±0.25^c^ | 6.00±0.00^c^ | 6.90±0.10^b^ |
| A | 1 | 8.80±0.00 | 8.80±0.00 | 6.50±0.30^B^ |
|  | 15 | 8.13±0.01 | 7.54±0.12 | 6.69±0.02 |
|  | 30 | 6.00±0.00 | 5.75±0.57 | 4.75±0.25 |
| B | 1 | 8.60±0.00 | 8.40±0.20 | 8.00±0.20^AB^ |
|  | 15 | 7.94±0.06 | 7.76±0.09 | 7.38±0.04 |
|  | 30 | 5.75±0.55 | 6.60±0.40 | 6.00±0.60 |
| C | 1 | 8.60±0.00 | 8.20±0.00 | 6.90±0.10^AB^ |
|  | 15 | 8.11±0.11 | 7.80±0.08 | 7.72±0.28 |
|  | 30 | 5.60±0.60 | 5.75±0.55 | 5.75±0.25 |
| D | 1 | 8.60±0.00 | 8.60±0.00 | 7.10±0.30^AB^ |
|  | 15 | 7.67±0.10 | 7.80±0.08 | 7.58±0.14 |
|  | 30 | 6.35±0.15 | 6.50±0.55 | 5.50±0.50 |

K: Control sample (not containing garlic), A: Yogurt containing Ankara garlic, B: Yogurt containing Mersin garlic C: Yogurt containing Maraş garlic, D: Yogurt containing Taşköprü garlic. The different lowercase letters in the same column indicate significant differences during storage period (P<0.05). The different uppercase letters in the same column indicate significant differences among the samples (P<0.05) SE:Standard error
